# Geographic stomatitis: An enigmatic condition with multiple clinical presentations

**DOI:** 10.4317/jced.55758

**Published:** 2019-09-01

**Authors:** Juliana-de Noronha-Santos Netto, Marielle-de Campos Dias, Thais-Roberta-Ura Garcia, Simone-de Macedo Amaral, Águida-Maria-Menezes-Aguiar Miranda, Fábio-Ramôa Pires

**Affiliations:** 1 Preceptor, Stomatology service – Brazilian Dental Association, Rio de Janeiro/RJ, Brazil and Preceptor, Oral Diagnosis service - Hospital Naval Marcílio Dias, Rio de Janeiro/RJ, Brazil; 2 Post-graduate student in Dermatology – Hospital Naval Marcílio Dias, Rio de Janeiro/RJ, Brazil; 3 Preceptor, Stomatology service - Brazilian Dental Association, Rio de Janeiro/RJ, Brazil

## Abstract

Geographic stomatitis is an uncommon inflammatory condition of unknown etiology. It is characterized by reddish areas surrounded by white borders affecting any location in the oral cavity and presenting a migratory and cyclic pattern. The most common affected sites include buccal mucosa, labial mucosa and mucobuccal fold. Some patients can complain of pain or burning sensation. There are few reports in the literature about this entity and its relationship with other oral and cutaneous conditions such as fissured tongue, Reiter’s syndrome, atopy and psoriasis has been suggested but it is still controversial. In the present study we describe three cases of geographic stomatitis associated with fissured tongue. Lesions involved the buccal mucosa, labial mucosa, soft palate and mucobuccal fold and all cases were diagnosed based on their clinical features. All patients were oriented about the innocuous behavior of the condition and were advised to avoid exposure of the lesions to irritation factors. The three presented cases highlighted the importance of a detailed oral mucosal examination by clinicians and provided further information about the natural history and clinical presentation of geographic stomatitis.

** Key words:**Geographic stomatitis, geographic mucositis, geographic tongue.

## Introduction

Geographic stomatitis (GS) (or Migratory stomatitis) is an uncommon oral condition that was first described and recognized as “Eritema migrans” in 1955 (1). Since then, multiple terms have been used, such as ectopic geographic tongue, stomatitis areata migrans or eritema migrans circinatum, but GS and migratory stomatitis are the most commonly used designations (2). GS is considered an inflammatory condition with psoriasiform pattern located at any site of the oral cavity, with unknown etiology. Clinical aspect shows red patches circunscribed by a thin slightly elevated white or yellowish border, with a migrating pattern and cyclic remission and reactivation (1-3). The most common affected sites include buccal mucosa, labial mucosa and mucobuccal fold (4). GS is more common in men and in adults and most cases are asymptomatic, but some patients can complain of pain or burning (2). Diagnosis is based on clinical examination, symptoms and natural history of the condition, but if clinical presentation is not typical and other chronic conditions are considered in the differential diagnosis, a biopsy is recommended. The term “Geographic tongue” describes the same psoriasiform mucositis affecting only the tongue (mostly the dorsum); contrarily to GS, geographic tongue is a very common condition, affecting up to 3% of the population (3). A few authors have suggested that geographic tongue could be associated to other systemic conditions (2,3,5), but this association has not been described in GS.

## Case Reports

-Case 1

**Figure 1:**
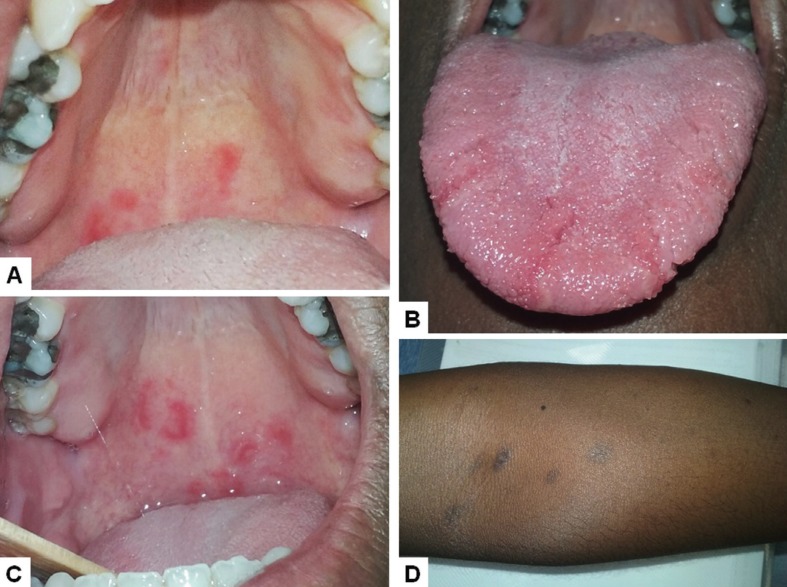
Case 1. Reddish patches in the soft palate in july 2017 (A); geographic and fissured tongue in July 2017 (B); slight changes in the clinical pattern of the palatal lesions in August 2017 (C); purplish polygonal patches diagnosed as cutaneous lichen planus affecting the left forearm (D).

A 33 year-old male, with previous diagnosis of cutaneous lichen planus was referred to evaluation of oral painless erythematous areas detected during dermatological routine examination. Intraoral examination showed multiple reddish patches circumscribed by a discrete whitish border in the soft palate and well-defined reddish patches limited by yellowish-white borders in the dorsum of the tongue and fissured tongue (Fig. 1A,B). The patient was under prednisone and griseofulvin use for management of the cutaneous lichen planus (Fig. 1D). Clinical diagnosis was GS and fissured tongue. Patient was oriented about the nature of the condition and also to avoid spicy and citric foods/beverages. No additional medications were prescribed. Clinical aspect one month later showed a change in the pattern (Fig. 1C), reinforcing the diagnosis of GS.

-Case 2

**Figure 2:**
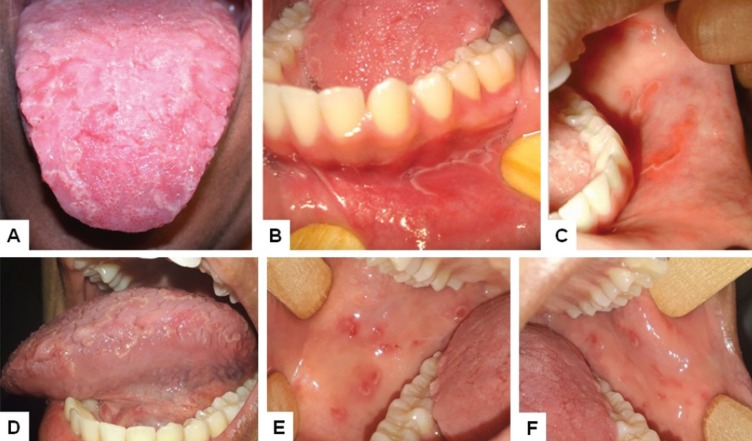
Case 2. Geographic and fissured tongue (A) and reddish patches circumscribed by a whitish halo in the labial (B) and buccal mucosa (C) in 2010; geographic and fissured tongue affecting the left border (D) and reddish patches with whitish halo in the right and left buccal mucosa in 2018 (E and F).

A 14 year-old female was referred for evaluation of an intraoral burning sensation lasting few months. Medical history revealed no past/present local or systemic mucocutaneous disorder. Intraoral examination showed fissured tongue associated with extensive reddish well-defined patches surrounded by a whitish irregular border affecting the tongue and also the lower labial mucosa, muccobuccal fold and buccal mucosa (Fig. 2 A,B,C). Clinical diagnosis was GS and fissured tongue. Patient was oriented about the nature of the condition and also to avoid spicy and citric foods/beverages. Due to the burning sensation, oral steroid rinses (5 mL dexamethasone, 3 times/daily, for 15 days) were prescribed with good response. The patient is under clinical follow-up for 8 years, showing episodes of cyclic onsets and eventual mild burning symptoms (Fig. 2D,E,F).

-Case 3

**Figure 3:**
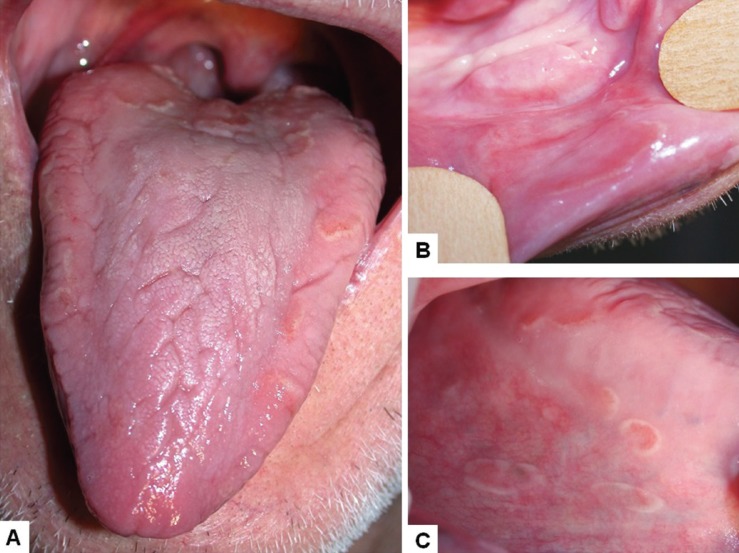
Case 3. Geographic and fissured tongue (A) and reddish patches circumscribed by a whitish halo in the lower labial mucosa (B); detail of the lesions in the right border of tongue (C).

A 70 year-old male was referred for evaluation of a burning sensation in the mouth lasting a few months. Medical history revealed mild arterial hypertension controlled with losartan, moderate alcohol consumption and no past/previous history of any mucocutaneous disorder. Clinical oral examination revealed fissured tongue and reddish patches surrounded by whitish irregular borders in the tongue, and buccal and labial mucosa (Fig. 3A,B,C). Clinical diagnosis was GS and fissured tongue. Patient was oriented about the nature of the condition and also to avoid spicy and citric foods/beverages. Due to the burning sensation, oral steroid rinses (5 mL dexamethasone, 3 times/daily, for 15 days) were prescribed to relief the symptoms. Unfortunately, patient was lost to follow-up.

## Discussion

Although GS can affect all anatomical locations in the oral cavity, the gingiva, hard palate and floor of mouth seems to be the less affected sites (6,7). When symptomatic, topical anesthetics, antihistamines, anxiolytics or topical steroids can be prescribed (2,4,6). Two out of the three included cases in the present series reported some discomfort in the oral cavity but required no medication. GS affect no particular age group and most studies suggest a male predominance and the present reported cases reinforce these features (7).

Diagnosis of GS relies on clinical signs and symptoms and natural history of the condition, as shown by the presented cases (2,6). However, if the pattern of the lesions is not typical and other chronic conditions, such as candidiasis or lichen planus could not be excluded, then a biopsy should be performed (6). Most cases reported in the literature showed that concomitant tongue involvement, in the form of conventional geographic tongue, is very common (2,7). In contrast to GS, geographic tongue is very common and is characterized by the isolated tongue involvement (8). Patients affected by geographic tongue can be also affected by fissured tongue (3,8,9). The three GS affected patients from the present study also showed fissured tongue.

The exact etiology of GS and geographic tongue is still unknown, but the latter has been associated with some oral and cutaneous conditions, such as fissured tongue (as seen in the present cases), Reiter’s syndrome, atopy and psoriasis (3,4,7,8). Some studies have previously suggested that geographic tongue can be associated with psoriasis based on clinical, histological, genetic (HLA associations) and epidemiological evidences (8). GS may also show both clinical and histological features resembling psoriasis. Picciani et al. (5) reported a case of GS in a 37 year-old female and suggested a histological and immunohistochemical similarity between this condition and psoriasis (5). Espelid et al. (2) reported 6 patients affected by GS, one of them presenting pustular psoriasis. Curiously, this patient was the only one manifesting oral lesions in the gingiva and hard palate, two unusual anatomical locations for GS. In contrast, Van der Wal et al. (7) studied 70 patients with psoriasis and found no patient with GS. None of the present patients reported a previous history of psoriasis, but interestingly one of the patients had a previous diagnosis of cutaneous lichen planus. Further collaborative studies are desirable to understand the possible relationship of GS with other oral and cutaneous diseases, especially psoriasis.

According with Hume (10), geographic tongue and GS can be classified in 4 subtypes ( Table 1) and the three patients included in the present series could be classified as type 2. Based on this background and the recent evidences that these conditions can be associated to mucocutaneous disorders (especially psoriasis) we propose a new classification based on both clinical aspect of the lesions and medical history of the patients ( Table 1). This new suggested classification updates Hume´s classification and reinforces the existence of GS, not only geographic tongue. This new suggested classification also highlights the possible association of GS to mucocutaneous and/or other systemic diseases (e. g. psoriasis and lichen planus), such as demonstrated by case 1. According with the present classification case 1 would be classified as type 6 and cases 2 and 3 as type 3.

**Table 1:**
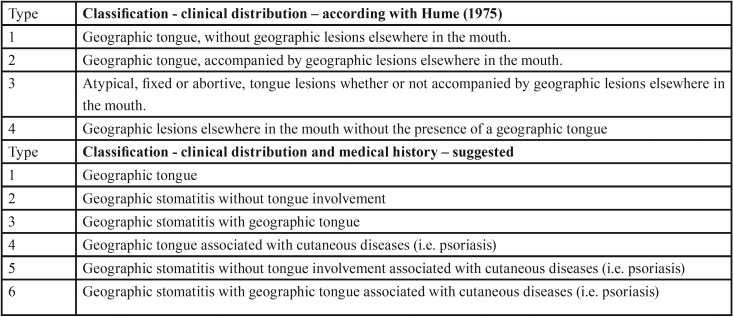
Classification of geographic tongue and geographic stomatitis, according with Hume (1975) (10) and the new suggested classification.

In conclusion, it is important that oral clinicians and medical professionals are aware of oral mucosal conditions that can be potentially associated with mucocutaneous diseases, both for early diagnosis and prompt referral of the patient to an Oral Medicine service when necessary. GS is a rare condition that can be under diagnosed or misdiagnosed in daily practice. Additionally it is possible that, similarly to geographic tongue, GS can be associated with systemic and mucocutaneous disorders, such as psoriasis. The present report suggested that the co-existence of GS and geographic tongue with systemic and mucocutaneous disorders should be always reported to provide information on their possible association. A modified classification based on Hume´s classification and including the above mentioned parameters was also suggested.
